# Noctopus: a novel device and method for patient registration and navigation in image-guided cranial surgery

**DOI:** 10.1007/s11548-024-03135-w

**Published:** 2024-05-15

**Authors:** Yusuf Özbek, Zoltán Bárdosi, Wolfgang Freysinger

**Affiliations:** Medical University of Innsbruck, University ENT Clinic, Innsbruck, Austria

**Keywords:** Patient registration, Fiducial configuration, Surgical navigation, Magnetic tracking, Optical tracking

## Abstract

**Purpose:**

A patient registration and real-time surgical navigation system and a novel device and method (Noctopus) is presented. With any tracking system technology and a patient/target-specific registration marker configuration, submillimetric target registration error (TRE), high-precise application accuracy for single or multiple anatomical targets in image-guided neurosurgery or ENT surgery is realized.

**Methods:**

The system utilizes the advantages of marker-based registration technique and allows to perform automatized patient registration using on the device attached and with patient scanned four fiducial markers. The best possible sensor/marker positions around the patient’s head are determined for single or multiple region(s) of interest (target/s) in the anatomy. Once brought at the predetermined positions the device can be operated with any tracking system for registration purposes.

**Results:**

Targeting accuracy was evaluated quantitatively at various target positions on a phantom skull. The target registration error (TRE) was measured on individual targets using an electromagnetic tracking system. The overall averaged TRE was 0.22 ± 0.08 mm for intraoperative measurements.

**Conclusion:**

An automatized patient registration system using optimized patient-/target-specific marker configurations is proposed. High-precision and user-error-free intraoperative surgical navigation with minimum number of registration markers and sensors is realized. The targeting accuracy is significantly improved in minimally invasive neurosurgical and ENT interventions.

## Introduction

Patient-to-image registration is the key aspect in minimally invasive image-guided interventions for neurosurgery or ENT specialties and significantly influences accurate surgical navigation. The decisive factors for the targeting accuracy of the intraoperative navigation [1, 2] are dependent on the applied registration techniques together with placement, distribution, correct detection and the number of registration markers/sensors, attached on the patient and utilized for intraoperative patient registration [3, 4]. In this process to couple the intraoperative physical patient’s anatomy with preoperative patient’s image datasets (e.g. CT, MRI, PET, SPECT, CBCT), sensors from optical or electromagnetic tracking systems and eventually, radiolucent markers placed on the patient, are used. To increase the patient safety and thus success rate of the intraoperative instrument alignment in the intracranial space, commercially available marker- and surface-based registration methods [5, 6] are applied state of the art in today’s clinical routines, jointly applicable with both tracking systems.

In both registration methods, to reach a minimum TRE and thus to realize a precise image-guided intervention, registration markers should be determined and marked as precisely as possible both in the virtual image dataset and on the patient. In addition, these markers should have their centre of mass (centroid) as close as possible to the operating area (target), be distributed spherically around it and/or be placed very close to it and not arranged collinearly [7]. According to the given requirements and literature, the invasive fiducial screw method allows a precise registration and accurate alignment of virtual and real patient anatomy, thus most accurate TRE. However, several registration screws are to be placed at discrete locations and spread empirically around the head, leading to large deviations of the TRE at different anatomical targets and preventing a robust and larger accuracy zone. The average intraoperative targeting accuracy using that procedure is given between 0.67 and 2.11 mm [8–11], while for dental splint between 1.0 and 4.9 mm [6, 11, 12], for anatomical landmarks 3.1 and 9.3 mm [13–17] and for skin adhesive registration method 0.8 and 3.8 mm [18–20] are presented in previous works. Screws do not change their position between preoperative imaging and surgery; patient’s soft tissues may change due to various reasons and severely affect surface registration approaches (1.3–5.35 mm for surface recognition [21, 22], 1.8–2.8 mm for laser-based [23, 24], 1.8–4.9 mm for pointer-based [12, 25] and 2.2–3.6 mm for LED mask-based [26, 27]) and have a large influence at the TRE. As with any invasive procedure, the attachment of the screw fiducials could carry the risk of infection, damage to anatomical structures and potential secondary bleeding or scars.

This paper describes a novel device and method to automatically determine the best placement positions of registration markers and their distribution in image-guided head surgery. The patient-/target-specific approach is independent of tracking technology and provides a robust and submillimetric targeting accuracy on all anatomical single or multiple target/s in the neurocranium for intraoperative surgical navigation. The device contains four registration markers on the rotatable and repositionable arms; it is attached onto the patient’s parietal bone preoperatively and scanned with the patient. After imaging, localized markers are used to generate marker configurations candidate for a specific target in the anatomy. A brute-force search method finds the best possible configuration by measuring the TRE for each candidate position preoperatively. The arms of the device allow positioning the sensors of a tracking system mechanically to the positions determined by the method around the head intraoperatively. In addition, surgeons obtain visual feedback of the expected TRE. The system provides means to reconfigure marker positions based for different target/s and clinical requirements, without additional imaging. This approach significantly increases the targeting accuracy for neurosurgical and ENT interventions, while possible disadvantages of invasive fiducial screw method are minimized.

## Materials and methods

This section describes the hardware and software components and the procedural methods of the presented system, respectively.

### Noctopus device

**Fig. 1:**
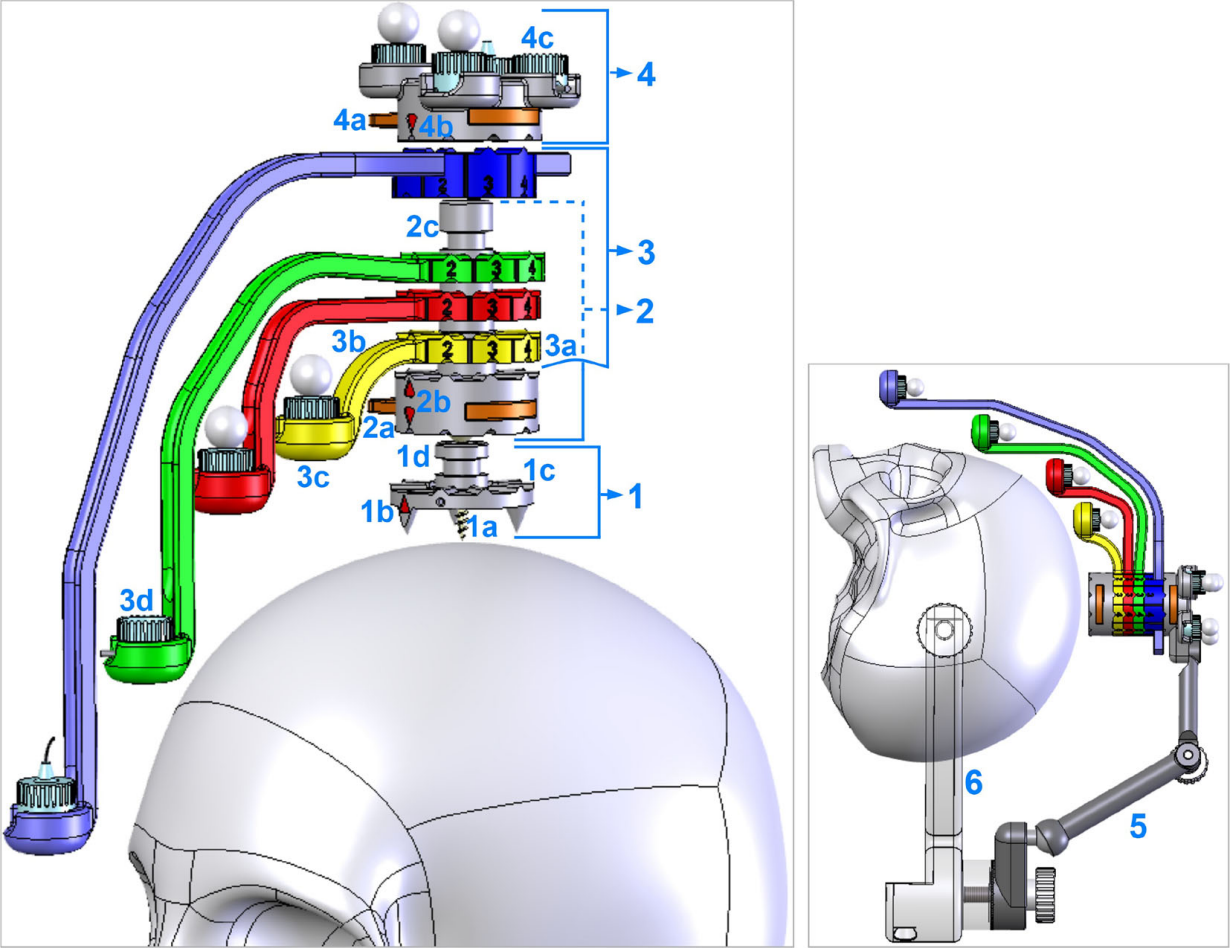
Noctopus device main and subcomponents with invasive and non-invasive use cases. (1) Patient base plate with a guide bore for electromagnetic 6-DOF reference frame sensor. (2) Collector (axis centred on the patient base plate) with a left–right slide lock mechanism (orange components). (3) Four marker wheels (blue, green, red and yellow) with corresponding numerals (3a), arms (3b) and marker/sensor holder at the end of each arm (3c) that can carry optical active, passive or electromagnetic sensors in the sensor holders (3d), respectively. (4) Reference frame plate (DRF) with slide lock and four sensor holders for sensors of optical tracking system. The overall device is a lightweight 3D-printable material with size of 55 mm x 35 mm excluding the arms

The head-mounted frameless stereotactic Noctopus device consists of CT/MRI compatible and biocompatible components (Fig. 1). The determination of the best possible registration marker configuration with a minimum TRE, takes place in three steps.

**Fig. 2:**
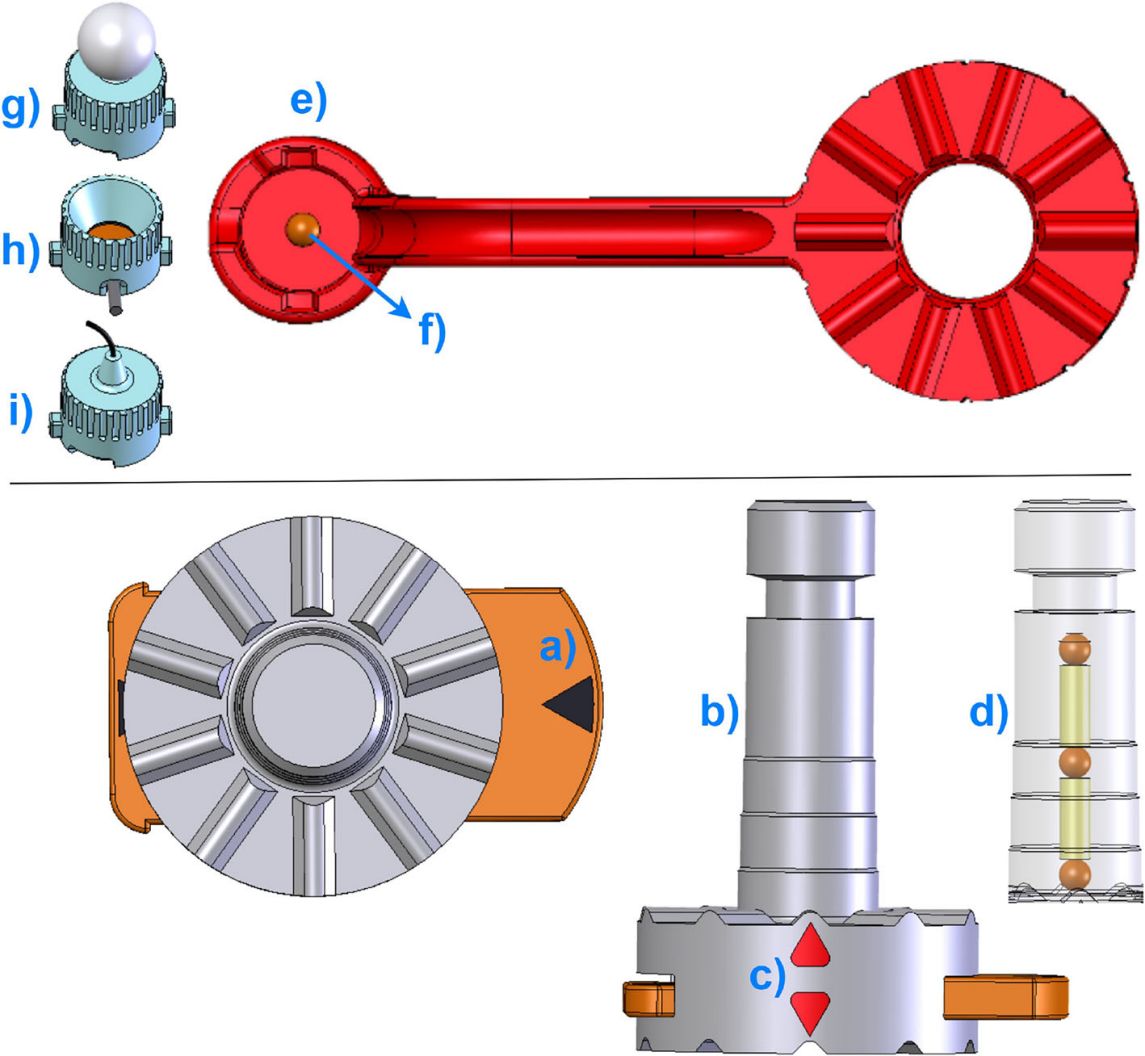
Top: Overview of one of the marker wheels with corresponding arm and attachable sensors on the MSH. Bottom: Views of the collector: Left and right show top and frontal views, respectively

**Fig. 3:**
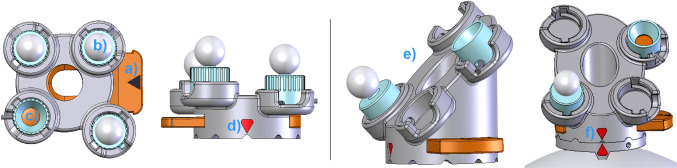
Top and front view of different DRF plates and its optional coupling with the PBP

**Fig. 4:**
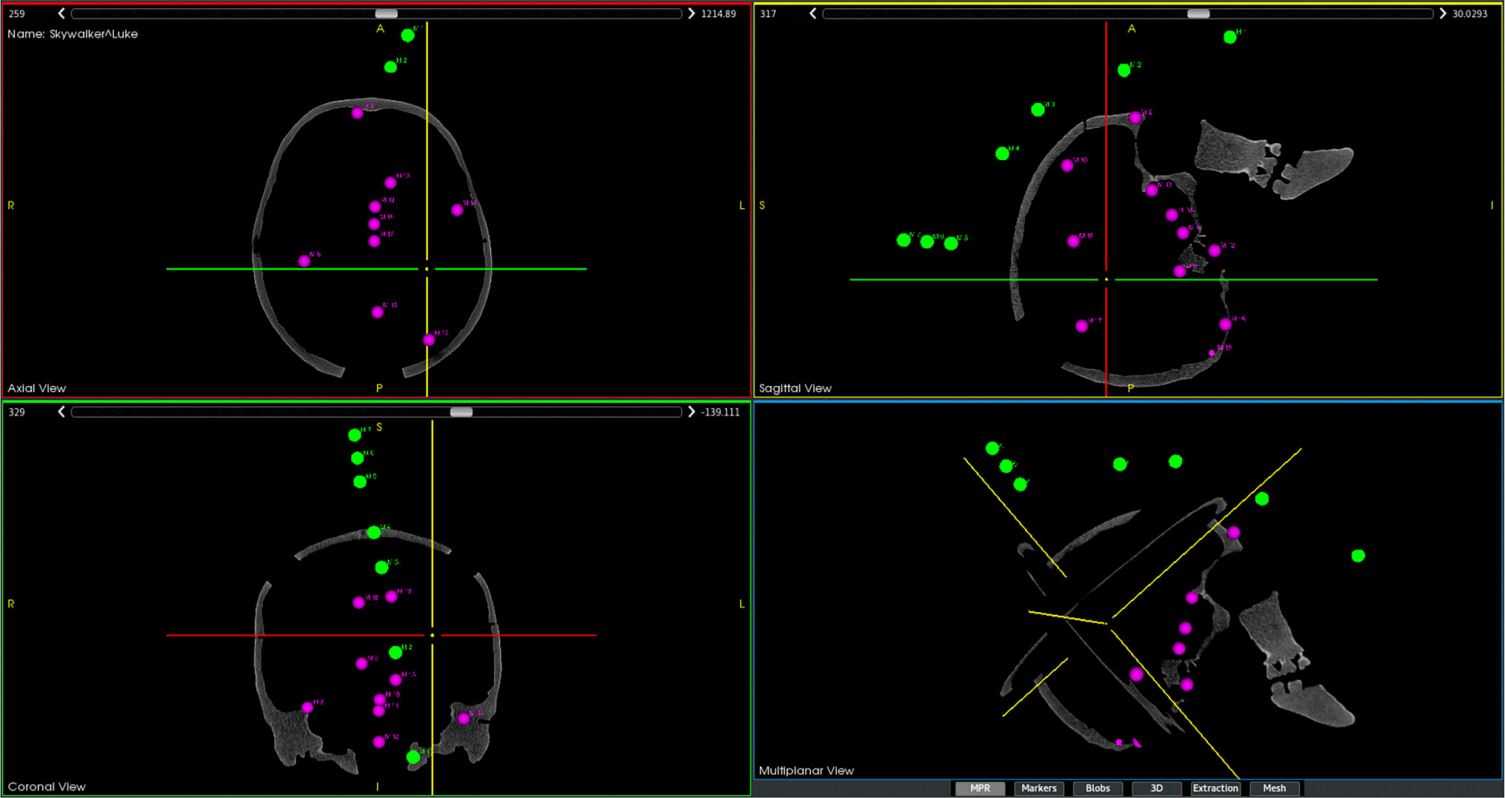
3D centroid positions of the localized three markers in the column and the four registration markers (green) and titanium screw targets (pink) in the patient’s anatomy, shown as axial, sagittal, multiplanar and coronal views. Clockwise from 4th quadrant

**Fig. 5:**
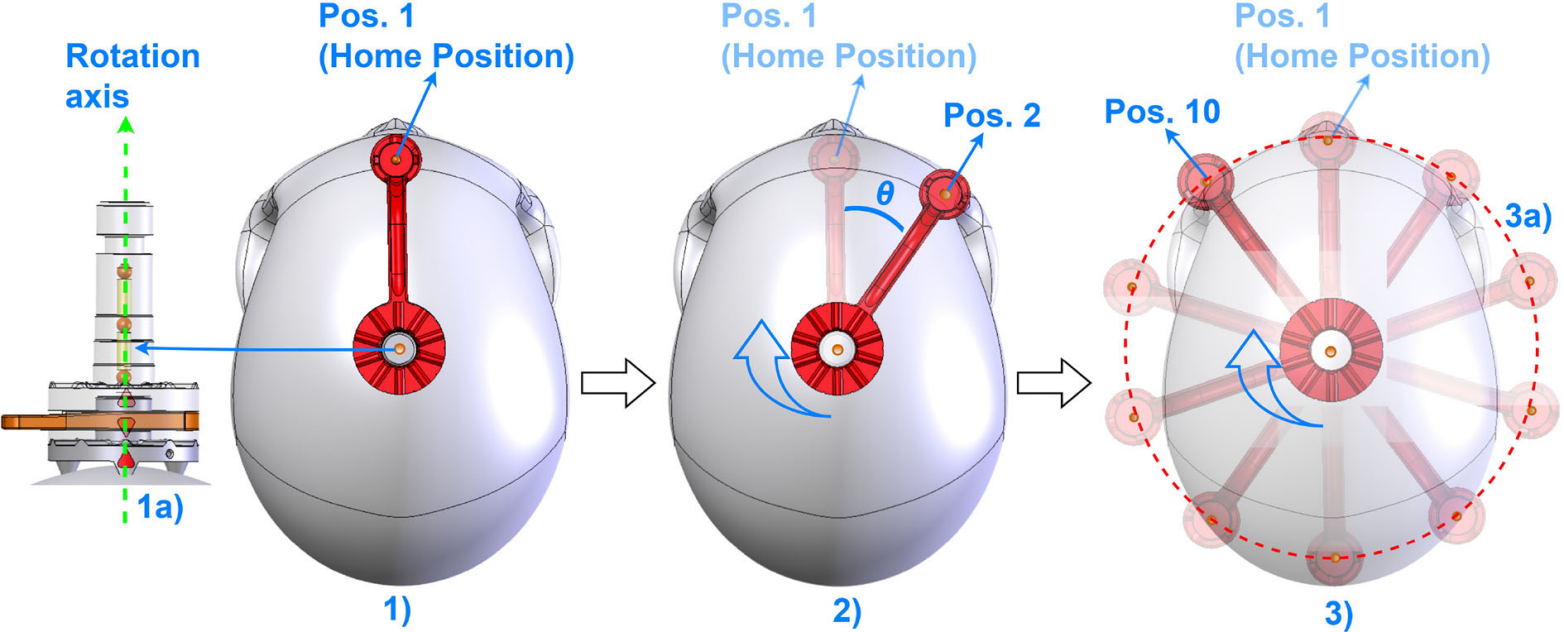
Stepwise generation of candidate registration markers. (1a) Representation of localized three markers in the column with a calculated 3D direction vector passing through the marker centroids (dashed line with the arrow) and a registration marker (1) in the MSH, while in home position, perpendicular to the vector (1a). (2) Representation of a single virtual rotation (Pos. 2, one step or 36$^{\circ }$ in clockwise direction) of a registration marker around the calculated rotational axis with an angle $\theta$. (3) Representation of possible ten rotational positions (registration marker candidates) of a single registration marker, located in the image dataset in its home position. The virtual positions are identical to the mechanical rotational positions of the marker wheels. The diameter of a rotation (3a, red dashed circle) is determined by the distance between the axis direction vector and 3D marker position in the MSH

**Fig. 6:**
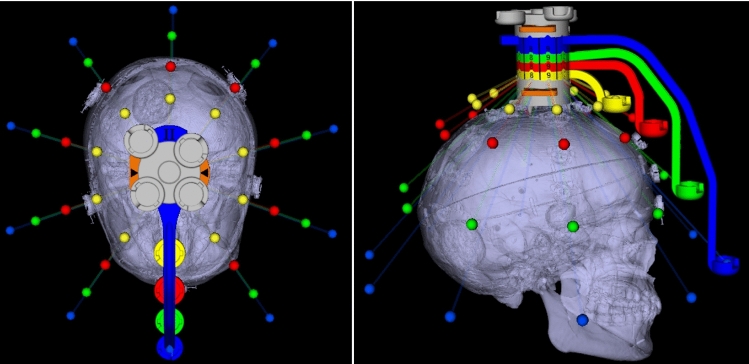
Generated possible candidate registration marker positions (color-coded, depending on the marker wheels) with their rotational axes on the collector. The rotation axis of the arms of the 3D model of the device is positioned on the parietal bone at the calculated rotation axis. Superior and right lateral views of 3D segmented patient dataset, prior to final registration marker configuration selection

The placement of the Noctopus device on the head is accomplished preoperatively either using the patient base plate (PBP) by anchoring it to the parietal bone e.g. vertex or bregma with a single self-cutting titanium bone screw (L: 9 mm, ${\oslash }$ 1.7 mm) (Fig. 1-1a) or using an articulated arm (Fig. 1-5), it is connected non-invasively and without a PBP to a standard Mayfield head clamp (Fig. 1-6). The PBP remains in situ until the surgical procedure is completed. The centred short cylindrical column (Fig. 1-1d) couples the PBP with either collector or DRF plate (optional) components. Coupling and decoupling of those components is realized with a left–right slide lock, which are integrated at the bottom side of collector and DRF plate (Fig. 1-2a and 4a). An electromagnetic 6-DOF sensor (L: 9 mm, ${\oslash }$ 0.8 mm) can be attached on the PBP through a guide bore for electromagnetic tracking. The indicators (Fig. 1-1b, 2b and 4b) show the mechanical positions of the marker wheels on the collector or DRF plate after coupling with the PBP. Mechanical positioning of all components on the PBP is ensured by a 10 teeth Hirth joint that mesh together on the end faces of each half shaft (Fig. 1-1c). Those radially arranged semi-cylindrical teeth, also on the collector, marker wheels and DRF plate, reliably define the possible mechanical angular positions of the four marker wheel arms.

The collector is coupled with the PBP via a slide lock (Fig. 2a, in opened position) and consists of a stepped cylindrical column (Fig. 2b) that prevents mis-stacking of the marker wheels and contains three axially fixed spherical CT/MRI compatible markers inside (Fig. 2d, transparent view of the column, each marker ${\oslash }$ 4 mm). All marker 3D positions are detected in the patient image dataset to define the rotation axis of the four marker wheel arms. The indicators (Fig. 2c) show the actual mechanical positioning of marker wheels on the collector. Four color-coded marker wheels (each H: 8 mm, ${\oslash }$ 35 mm) with their arms, each one having a radiolucent spherical marker at a well-defined position, are stacked on the rotation axis (Fig. 2f, ${\oslash }$ 4 mm) to be localized in the patient dataset, in the marker/sensor holders (MSH) at the end of an individual arm (Fig. 2e). Each arm is of different length, designed for up to 66 cm head circumference and kept as close as possible to the patient’s anatomy, so that the whole head is covered. They can carry an optical passive reflective sphere, an active infrared LED or an electromagnetic sensor (Fig. 2g,h,i) in its sensor holder (SH). The rotations or mechanical positioning possibilities (registration marker configuration) of an individual marker wheel around the collector are determined by ten numerals (between one and ten steps) using Hirth-toothings and may be varied in steps of 36$^{\circ }$.

When an electromagnetic tracking system is used during the intervention, the DRF plate is rigidly attached on the collector (Fig. 3a, in locked position) only for fixation purpose of the marker wheels. When an optical tracking system is intended, it also carries four optical active or passive sensors on it (Fig. 3 shows three passive, (b) and one active attached sensors, (c)). Depending on the surgery and physical position of the optical tracking system in the operating room, the DRF plate can be designed in a vertically (Fig. 3 left) or in a horizontal/oblique orientation (Fig. 3e right). During patient imaging, all marker wheels are positioned in position 1 (home position) as shown in (Fig. 1). After the registration marker configuration and intraoperative patient registration, but before the surgical navigation, the collector thus also the marker wheels can be decoupled from the PBP and the DRF plate can be coupled with the PBP (Fig. 3f) to allow an unobstructed surgery.

### Marker detection and localization

A phantom with twelve anatomical targets, realized through implanted titanium bone screws (head ${\oslash }$ 2 mm) and an attached Noctopus device with marker wheels positioned in home position was scanned in HFS position using a CT scanner at the University Clinic for Radiology in Medical University of Innsbruck. The CT image dataset had a slice thickness of 0.6 mm, with a resolution of 512x512 pixels. It consisted of 504 slices, each with a pixel spacing of 0.488 mm x 0.488 mm. The dataset was loaded into the Noctopus navigation software and visualized as standard DICOM view (Fig. 4), without undergoing any reconstruction or post-processing. The centroids of all registration markers in the MSHs of each particular marker wheel arm and markers in the collector’s column were detected, and their 3D positions in image space were localized automatically based on their geometrical properties using morphological operations [28]. The localized positional coordinates of the markers in the column were used to determine the spatial direction (unit) vector of the rotational axis of marker wheels, while the registration markers in the MSHs serve to generate the 3D candidate registration marker positions around the patient’s head.

### Determining the rotational axis and arm positions

To match mechanical (in the operating room) and virtual (in the imagery) of marker wheel coordinates the 3D rotation axis of the rotatable arms, mechanically identical to the centre of the column, is identified. The axis is found as the line passing through the centroid positions of three collinear markers, in the least squares sense (Fig. 5).

**Fig. 7:**
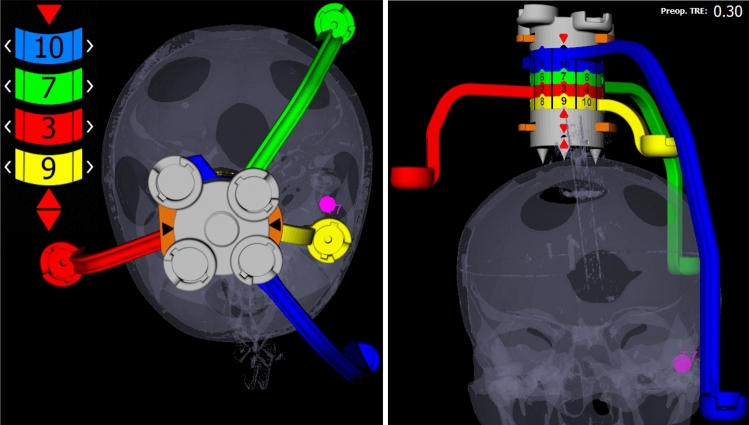
Marker configuration for a desired target (pink sphere) inside the patient’s 3D model, represented from superior and anterior directions. The blue marker wheel is set to the position number 10, while green to 7, red to 3 and yellow to 9 as indicated on the marker wheels by the indicators and additionally shown as enlarged at the left side of the scene. Synthetic radiograms (cranio-caudala, left and frontal, right) with overlay of Noctopus device

**Fig. 8:**
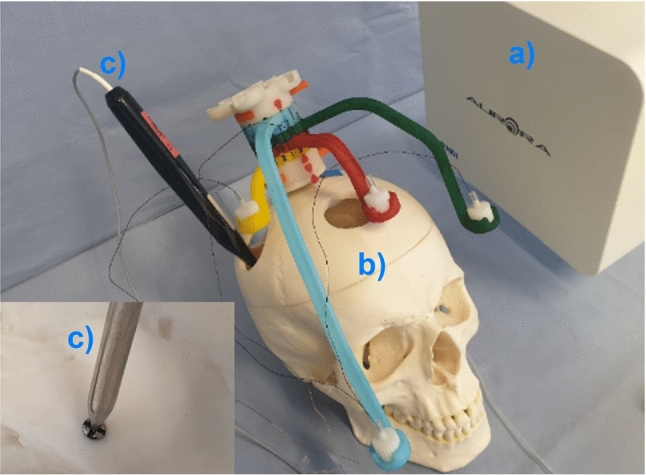
Experimental setup with 3D-printed Noctopus device. **a** Aurora tracker. **b** Navigated phantom patient with attached and configured Noctopus device. **c** Navigated probe placed on one of the target screw head

The possible candidate rotational positions subset $S_i(x',y',z'), \left| S_i \right| = 10$ of a localized single registration marker (Fig. 6) is generated using the rotation matrix: $$\begin{pmatrix} x' \\ y' \\ z' \end{pmatrix}=\begin{bmatrix} (p(j^2\text {+}k^2)-i(qj\text {+}rk-ix-jy-kz))(1-c)\\ \quad \text {+}xc\text {+}(-rj\text {+}qk-ky\text {+}jz)s \\ (q(i^2\text {+}k^2)-j(pi\text {+}rk-ix-jy-kz))(1-c)\\ \quad \text {+}yc\text {+}(ri-pk\text {+}kx-iz)s \\ (r(i^2\text {+}j^2)-k(pi\text {+}qj-ix-jy-kz))(1-c)\\ \quad \text {+}zc\text {+}(-qi\text {+}pj-jx\text {+}iy)s \end{bmatrix}$$

where $(i, j, k) \in \mathbb {R}^{1 \times 3}$ is direction vector of rotational axis, $(p, q, r) \in \mathbb {R}^{1 \times 3}$ is the pivot point of the direction vector passing through the marker centroids in the column, $(x, y, z) \in \mathbb {R}^{1 \times 3}$ is home position of a registration marker in image space, *c* is *cos* $\theta$, and *s* is *sin* $\theta$. $\theta$ is varied in steps of 36$^{\circ }$.

**Fig. 9:**
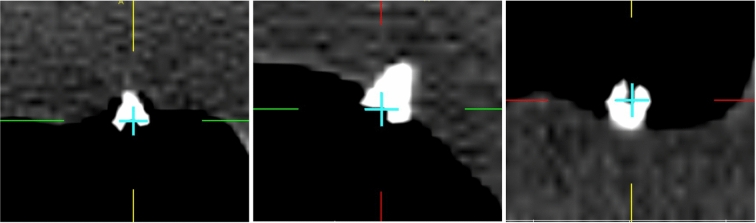
Partial screenshot of the intraoperative navigation while the accuracy of the registration on a target was qualitatively evaluated by localizing the implanted titanium screws with the navigation probe. The crosshair on DICOM views (axial, sagittal and coronal, from left to right) indicates the actual position of the probe tip placed on the screw head. Small titanium screws are known to hardly produce image reconstruction artefacts in CT imaging [30]; thus, the clarity of the images is not affected

**Table 1:** Intraoperative targeting accuracy results in mm for various anatomical targets in the cranial space using determined best marker configurations

Surgical target	Marker configuration	FRE	FLE	TRE	*d* CoM to target
	B, G, R,Y				
1- Cochlea	3, 6, 10, 4	0.42	0.35	0.19	49.86
2- Anterior Fossa	2, 10, 1, 3	0.38	0.28	0.14	20.78
3- Sella Turcica	1, 4, 7, 9	0.50	0.50	0.27	47.68
4- Sphenoid Sinus	2, 9, 5, 6	0.45	0.40	0.23	53.64
5- Central Skull Base	2, 5, 9, 8	0.46	0.42	0.26	71.52
6- Optic Canal	10, 3, 7, 1	0.28	0.15	0.07	35.23
7- Middle Fossa	10, 7, 3, 9	0.48	0.46	0.27	65.05
8- Posterior Fossa	6, 9, 3, 7	0.32	0.20	0.12	81.96
9- Foramen of Luschka	6, 3, 9, 8	0.52	0.54	0.34	82.17
10- Optic Lobe	6, 4, 7, 2	0.46	0.42	0.21	4.63
11- Parietal Lobe	3, 10, 7, 5	0.56	0.62	0.31	10.70
12- Frontal Lobe	10, 3, 1, 8	0.53	0.56	0.28	14.88
Mean/SD		0.44 ± 0.08	0.40 ± 0.14	0.22 ± 0.08	

**Fig. 10:**
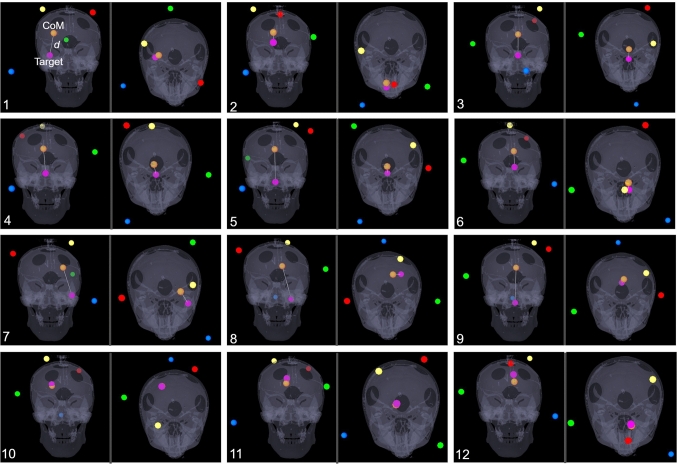
Determined best marker configurations for each individual target and distance between marker’s centroid (CoM, orange sphere) and target (pink sphere), represented from anterior and superior directions on the patients 3D model. The Noctopus device was removed from the scene, and only corresponding marker positions were left for better visibility. Spherical distribution and not collinear arrangement of the registration markers around the target are noticeably. Additionally, for off-centre targets (1, 2, 7, 8, 9, 10 and 12) a variety of registration markers are positioned near to the target, while one of them at a distance or contralateral, which provides a low, uniform TRE

### Determining the best registration marker configuration

A brute-force search finds the best configuration on base of all possible candidate positions $S= \left\{ S_{1},...,S_{n= 10^{4}} \right\}$ from all marker wheel permutations obtained in 2.3. For each permutation step $S_{i}$, a TRE value $(TRE_{i})$ is calculated [29] and the fiducial localization error is estimated ($FLE_{est}$) from repeated FRE measurements [29] as $\left\langle FLE_{est}^{2} \right\rangle =\frac{N}{N-2} \times \mu$, where *N* is the number of registration markers and $\mu$ is the squared average value of measured FREs, preoperatively. This process finds the four marker wheel positions with minimum TRE value, where $$TRE_{min} =min\begin{bmatrix} \begin{Bmatrix} TRE_{i} \end{Bmatrix} i= 1,...,i^{10^{4}} \end{bmatrix}$$ in the preoperative phase (Fig. 7). This can be also done intraoperatively for a single or multiple anatomical target/s. After rotating the marker wheels to the recommended configuration, the sensors of the selected tracking system are attached into the marker/sensor holders and the sensor positions read out. The patient is registered with the preoperative image dataset automatically using the standard rigid-body registration technique [3].

### Noctopus navigation software

A plugin-based, platform-independent surgical navigation software system featuring marker localization, pre-/intraoperative marker configuration, intraoperative patient registration and navigation was developed. All the required modules were implemented using open-source C++ and Python libraries such as the common toolkit, the visualization toolkit, insight segmentation and registration toolkit, image-guided surgery software toolkit and open network interface for image-guided therapy and runs on a standard computer.

## Evaluation

To quantify the impact of registration marker configuration on the targeting accuracy, the proposed system was evaluated under laboratory conditions using an electromagnetic tracking system (NDI Aurora V3, Northern Digital Inc., Canada) and an anatomic phantom skull. After imaging the phantom patient, the image dataset was loaded into the Noctopus software. Marker localization and configuration were applied for all available individual targets.

To determine the approximately expected intraoperative TRE and the best registration marker configurations for each target, the preoperative TRE using the configured marker and target screw positions in image space was measured. In order to estimate the FLE before measuring the TRE first, the patient was placed within the field of view of the electromagnetic tracking system, four 5-DOF sensors were then attached into the configured MSHs, while a 6-DOF DRF sensor was attached onto the PBP. The sensor positions were read out, and the patient was registered to the image dataset to measure the FRE. The patient registration for each target was repeated ten times and averaged. The obtained FREs were then used to calculate the FRE-based FLE and averaged.

To realize the real-time surgical navigation and measure the intraoperative TRE for each target, sensors were attached on the Noctopus as done in preoperative TRE measurement. The patient was registered with mechanically set marker configuration (Fig. 8) and the actual FRE was determined, respectively. The TRE was measured by using the positions of tracked sensors in each MSH and the probe position by placing its tip on the screw heads in the tracker space (Fig. 9), and 500 TRE measurements were obtained and averaged, respectively.

## Results

Table 1 gives the resulting targeting accuracies and standard deviations from the automatically determined best registration marker configurations during the evaluation for each given anatomical targets in the cranial space. The resulting marker wheel rotations were decided by a brute-force search from $10^4$ possible configuration positions by measuring the targeting accuracy for each configuration, respectively. The best TRE was observed where the centroid of configured markers coincides with the targets (*d* CoM to Target). The prototype system was able to reach an average submillimetric RMS FRE of 0.51 ± 0.15 mm, 0.44 ± 0.08 mm and TRE of 0.28 ± 0.02 mm, 0.22 ± 0.08 mm for preoperative and intraoperative measurements using configured marker wheel positions (B: blue, G: green, R: red and Y: yellow) determined from the proposed method, respectively. The overall accuracy decreases slightly when the 3D distance between the marker’s centroid and the target grows (Fig. 10). The overall run time to determine the best possible registration marker configuration took $\approx$ 2 sec, while it took less than 2 mins to establish surgical navigation, including marker localization and configuration, arrangement of marker wheels, sensor placement and patient registration.

## Discussion and conclusions

Presented novel device and method for automatized patient registration and surgical navigation using target-specific best possible marker configurations with precise correlation between diagnostic images and real patient anatomy, has shown submillimetric and very promising targeting accuracies in all anatomical regions of the neurocranium. The results confirm the rules [7] that the markers ideally surround the target, be distributed spherically around it, be not arranged collinearly, and the centroid of the marker configuration should coincide with the target. User-error-free, tracking system-independent, very fast and simple patient registration, configured consistently with a good marker arrangement using the required minimum number of sensors and disallowance of linear marker arrangement due to its mechanical construction and implemented method automatically, ensure constant, robust and reliable guidance during the intraoperative patient navigation. In particular, previous knowledge of the expected precision for the intervention preoperatively, facilitates trajectory planning, its execution in the intraoperative phase and reduces precision-dependent unexpected complications. In addition to anchoring of the small frameless device with only one bone screw a patient-friendly, non-invasive usage is possible that reduces the adverse events occurring in invasive fiducial screw registration technique.

Since the targeting accuracy is significantly increased and the resulting precision for single or multiple target/s overall in the patient’s anatomy is uniform and universally valid, the proposed system may allow to extend the use of navigation to new minimally invasive interventions in neurosurgery and ENT specialties that previously were not possible due to limitations in accuracy.
