# Correction to: Noctopus: a novel device and method for patient registration and navigation in image-guided cranial surgery

**DOI:** 10.1007/s11548-024-03251-7

**Published:** 2024-08-23

**Authors:** Yusuf Özbek, Zoltán Bárdosi, Wolfgang Freysinger

**Affiliations:** Medical University of Innsbruck, University ENT Clinic, Innsbruck, Austria

**Correction to: International Journal of Computer Assisted Radiology and Surgery** 10.1007/s11548-024-03135-w

The article Noctopus: a novel device and method for patient registration and navigation in image-guided cranial surgery, written by Yusuf Özbek, Zoltán Bárdosi and Wolfgang Freysinger, was originally published electronically on the publisher’s internet portal on 15 May 2024 without open access. With the author(s)’ decision to opt for Open Choice the copyright of the article changed on 16 July 2024 to © The Author(s) 2024 and the article is licensed under a Creative Commons Attribution 4.0 International License, which permits use, sharing, adaptation, distribution and reproduction in any medium or format, as long as you give appropriate credit to the original author(s) and the source, provide a link to the Creative Commons licence, and indicate if changes were made. The images or other third party material in this article are included in the article’s Creative Commons licence, unless indicated otherwise in a credit line to the material. If material is not included in the article’s Creative Commons licence and your intended use is not permitted by statutory regulation or exceeds the 123 International Journal of Computer Assisted Radiology and Surgery permitted use, you will need to obtain permission directly from the copyright holder. To view a copy of this licence, visit http://creativecommons.org/licenses/by/4.0/.

The original article has been corrected.

